# Adipose-derived stem cells alleviate radiation-induced muscle fibrosis by promoting muscle regeneration

**DOI:** 10.3389/fcell.2025.1620998

**Published:** 2025-10-24

**Authors:** Sha Li, Mingjing Peng, Xiang Ou, Zhijiao Zhou, Luyuan Xie, Yuxin Ge, Zehong Song, Xiao Zhou, Chunmeng Shi, Xiaowu Sheng

**Affiliations:** ^1^ Central Laboratory, Hunan Cancer Hospital and The Affiliated Cancer Hospital of Xiangya School of Medicine, Central South University, Changsha, Hunan, China; ^2^ Department of Endocrinology, The Affiliated Changsha Central Hospital, Hengyang Medical School, University of South China, Changsha, Hunan, China; ^3^ Department of Pathology, Third Xiangya Hospital, Central South University, Changsha, Hunan, China; ^4^ Changsha Medical University, Changsha, Hunan, China; ^5^ Department of Dermatology, The Second Xiangya Hospital, Central South University, Changsha, Hunan, China; ^6^ State Key Laboratory of Trauma, Burns and Combined Injury, Institute of Rocket Force Medicine, Third Military Medical University, Chong Qing, China

**Keywords:** radiation-induced muscle fibrosis, radiotherapy, adipose-derived stem cell, satellite cells, Apoptosis

## Abstract

**Background:**

Radiation-induced muscle fibrosis (RIF) is a severe late-stage side effect of radiotherapy in adjacent normal tissues, significantly affecting anticancer therapeutic efficacy and potentially being life-threatening. Previous studies have shown that satellite cells (SCs) become activated after ionizing radiation to facilitate muscle tissue repair. However, the acceleration and strengthening of this process have received little attention until recently. Adipose-derived stem cells (ADSCs), a type of mesenchymal stem cell, have emerged as a promising therapeutic option in regenerative medicine due to their accessibility, abundance, and plasticity in adult organisms. In this study, we explored whether ADSCs could enhance SC proliferation and differentiation after radiation therapy.

**Methods:**

ADSCs were harvested, cultured, and passaged from male Sprague–Dawley rats and characterized *in vitro*. *In vivo*, rats were randomly assigned to control and ADSC-treated groups (n = 6). ADSCs were transplanted into RIF rat models at different time points (4, 12, and 24 w). The therapeutic effects of transplanted ADSCs were assessed via Masson’s trichrome staining, electron microscopy, and hematoxylin–eosin (H&E) staining. SC activation, proliferation, and central nuclear immigration following ADSC transplantation therapy were evaluated via real-time polymerase chain reaction and H&E staining.

**Results:**

*In vivo*, fibrosis was markedly alleviated over time following ADSC treatment. In the RIF rat model, ultrastructural histopathological changes, including mitochondrial edema and vacuolization, myofilament dissolution, and vascular endothelial swelling, were notably attenuated by ADSC transplantation. Additionally, SCs exhibited a significant increase in activation and proliferation in the ADSC-treated groups, accompanied by a decrease in fibrotic symptoms.

**Conclusion:**

Our study provides evidence that ADSCs protect against RIF by promoting SC activation, proliferation, and differentiation *in vivo*. ADSCs may represent a promising therapeutic candidate for restoring muscle dysfunction and abnormalities caused by RIF.

## Introduction

Over the past several decades, the survival rates of cancer patients have significantly improved (Siegel et al., 2023; Lu et al., 2023). Radiation therapy remains a mainstay of cancer treatment (Chandra et al., 2021). However, exposure of the surrounding normal tissues to radiation often leads to serious side effects, resulting in functional impairments in cancer survivors (Palmer et al., 2021; Stokkevåg et al., 2024; Wang et al., 2024). Radiation-induced fibrosis (RIF) is a severe adverse effect of radiation therapy, especially in patients with head and neck malignancies (Lennox et al., 2002) or breast cancer (Van Geel et al., 2011). RIF primarily manifests as skin induration and thickening (Dancey and Waters, 2006; Yanaba et al., 2015), muscle weakness (Ghosh and Milone, 2015), atrophy (Jit et al., 2021), restricted mouth opening (Abboud et al., 2020), difficulty in eating and swallowing (Kawashita et al., 2022), and even respiratory failure (Lei et al., 2021). Several treatment options for RIF are currently available in clinical practice, including pentoxifylline (Okunieff et al., 2004; Binatti et al., 2021), sulforaphane (Wang et al., 2022), vitamin E (Krejbich and Birringer, 2022), glucocorticoids (Agha-Hosseini et al., 2021), and hyaluronic acid (Agha-Hosseini et al., 2021). However, the efficacy of these therapies remains limited, prompting the need for alternatives to mitigate this secondary injury. The underlying biological mechanisms of RIF are traditionally associated with DNA single- and double-strand breaks, inflammation (Chen et al., 2023; Vallée et al., 2017; Kiang et al., 2010), apoptosis, pyroptosis (Yu et al., 2021), and autophagy (Chaurasia et al., 2019). These processes directly or indirectly impair skeletal muscle function. Simultaneously, fibrosis reduces the regenerative capacity of skeletal muscle *in vivo*, resulting in sarcopenia and muscle contractile dysfunction (Gionet-Gonzales et al., 2023; Garg et al., 2015).

Adipose-derived stem cells (ADSCs) are multipotent mesenchymal adult stem cells derived from adipose tissue, and they are a promising therapeutic option for various diseases (Al-Ghadban and Bunnell, 2020; Alió Del Barrio et al., 2022). ADSCs are thought to possess robust biological potency of exocrine and paracrine functions (Harasymiak-Krzyżanowska et al., 2013), comprising factors such as the fibroblast growth factor (FGF) (Guo et al., 2022), vascular endothelial growth factor (VEGF) (Alió Del Barrio et al., 2022), transforming growth factor beta 1 (TGF-β1) (Ademi et al., 2023), and insulin-like growth factor 1 (IGF-1) (Li et al., 2019). The majority of these factors contribute to cell proliferation and neovascularization (Hu et al., 2020; Liao et al., 2024), especially preventing cell apoptosis (Song et al., 2024). Previous research revealed that ADSC-conditioned medium decreased the expression of apoptosis-related proteins in mice with myocardial infarction through the miR-221/222/p38/NF-κB pathway (Lee et al., 2021). Interestingly, Ai et al. found that ADSC transplantation could mitigate granulosa cell apoptosis in a rat model of premature ovarian failure (Ai et al., 2023). In addition, exosomes secreted from ADSCs ameliorated diabetic nephropathy complications by inhibiting podocyte apoptosis, thus improving outcomes *in vivo* (Jin et al., 2019). Moreover, ADSC transplantation promoted a wide range of anti-inflammatory cytokines in a multiple sclerosis murine model, including IL-6, IL-10, and TGF-β, thus preventing astrocyte activation and promoting the macrophage M2 phenotype (Al-Ghadban and Bunnell, 2020). Numerous studies showed that exosomes secreted by ADSCs consist of lipids, proteins, and miRNAs and are similar to their parental cells, whose antioxidant, anti-apoptotic, anti-inflammatory, and anti-fibrotic capabilities are evidently elevated in cardiovascular disease (Ren et al., 2024). In addition, cultured ADSCs isolated from adipose tissue have been shown to influence myofibroblast differentiation and can alleviate collagen accumulation, a process partly mediated by their paracrine functions in hypertrophic dermal scarring (Higginbotham et al., 2024). Furthermore, human platelet lysate-cultured ADSC sheets could significantly accelerate wound healing and mitigate macrophage recruitment while reducing subsequent wound tissue fibrosis in a burn wound Wistar rat model *in vivo* (Chen et al., 2024). Taken together, therapeutic intervention with ADSCs reduces cell apoptosis and promotes cell proliferation to ameliorate the damage caused to various tissues in early disease and fibrosis in the advanced state.

Satellite cells (SCs), which are unipotent stem cells, are responsible for postnatal skeletal muscle repair following various injuries (Zhang et al., 2024). They are generally present in a state of quiescence and are located in a specialized compartment (niche) between the basal lamina and myofiber sarcolemma (Zeng et al., 2022). SCs are activated in response to injury or altered muscle homeostasis and then divide asymmetrically in order to maintain self-renewal or form a proliferative population of myoblasts (Price et al., 2024). These committed precursors proliferate and differentiate into new myofibers, fusing with each other or with damaged fibers (Sampath et al., 2018). Paired gene 7 (Pax7) is expressed in the quiescent SC state as a unique marker in skeletal muscles (Diao et al., 2012). In contrast, the myogenic determination factor (MyoD) is not expressed in detectable levels in quiescent SCs, but it is upregulated early after activation (Yoshimoto et al., 2020). In the late 1980s and early 1990s, researchers discovered that myogenic factor 5 (Myf5) coordinates with numerous other myogenic regulatory factors, including myogenin (MyoG), participating in the regulation of the myogenic process (Brack and Rando, 2012). Although the nuclei are located at the center in most cells, the nuclei of normal muscle fibers lie peripherally near the cell membrane (Snijders et al., 2020). Nuclei centripetal migration in skeletal muscle cells can be observed only under certain specific conditions, such as during growth, regeneration, and pathophysiological processes (Snijders et al., 2020). According to Srikuea and Hirunsai, the central nuclei migration originating from SCs is a hallmark of muscle regeneration (Srikuea and Hirunsai, 2016).

In our previous research, we found that apoptosis plays an important role in radiation-induced dermatitis and fibrosis using an established dermatitis rat model (Sheng et al., 2019). Furthermore, we revealed that ADSCs alleviated radiation-induced dermatitis by suppressing apoptosis in a cathepsin F-dependent manner (Yao et al., 2021). More importantly, SCs are activated from the dormant state and undergo myogenic differentiation after ionizing radiation stimulation, but this is insufficient to combat fibrosis formation due to the low rate of muscle regeneration (Zeng et al., 2022). Based on this research, we inferred that ADSCs may similarly promote regeneration and inhibit muscle cell apoptosis in RIF. In this study, we first extracted ADSCs from adipose tissue and characterized them. Then, we treated an RIF rat model with ADSCs to evaluate the proliferation and regeneration of SCs *in vivo* and evaluated the inhibitory effect of ADSCs on apoptosis in irradiated muscle. In summary, we explored the potential therapeutic capability of ADSCs in RIF and its underlying mechanism preliminarily.

## Materials and methods

### Experimental animals

Two-month-old Sprague–Dawley (SD) rats procured from Hunan SJA Laboratory Animal Co., Ltd. (Hunan, China) were utilized in this study. All research members were formally trained in technologies and obtained relevant certification at Central South University before the operation. All the rats had free access to an irradiated chow diet and tap water for 7 days to acclimate to the new environment. Ethical approval and consent were obtained from the Animal Ethics Committee of Hunan Cancer Hospital, in accordance with the institutional guidelines for animal protocols.

### Establishment of RIF rat models and ADSC treatments

All 42 female rats were divided randomly into seven groups (n = 6), namely, normal, untreated control 90 Gy-4 w, 90 Gy-12 w, and 90 Gy-24 w, and ADSCs-treated 90 Gy-4w, ADSCs-90Gy-12 w, and ADSCs-90Gy-24 w groups. The normal group received only phosphate-buffered saline (PBS) injections. The radiation sites were the medial left thigh of the rat hind limb, which was clipped free of hair. After rats were anesthetized using 5% pentobarbital sodium, the untreated control groups received a single dose of 90 Gy irradiation and PBS injection, while the treated groups received 90 Gy irradiation and 10^7^ ADSC injections within 24 h, as a previous study reported (Yao et al., 2021). The detailed transplantation steps were as follows: first, the ADSCs were resuspended in 100 μL of PBS, divided into 25 μL per injection, and injected at a dose of 25 μL at each of the four points, i.e., above, below, to the left, and to the right of the radiotherapy site. At 4, 12, and 24 weeks after radiotherapy, all rats from each group were sacrificed under deep anesthesia.

### Isolation, culture, differentiation, and characterization of ADSCs

Subcutaneous adipose tissue was aseptically harvested from the groin region of male SD rats under anesthesia. After carefully removing large blood vessels, the tissue was rinsed three times with PBS (Gibco, Carlsbad, CA, United States) and then sheared into small fragments of 1 mm^3^. These fragments were digested with type I collagenase (1 μg/mL; Gibco) in a centrifuge tube and incubated in a thermostatic water bath at 37 °C for 1 h. Cell culture medium was added to the centrifuge tube to terminate the collagenase activity, and the disaggregated tissues were collected for centrifugation at 1,000 *g* at 25 °C for 10 min. The supernatant was aspirated and discarded using a pipette, and the cell pellets were filtered through a 70-μm stainless steel mesh to remove excess tissue clumps. The filtrate was then centrifuged again at 1,000 × *g* at 25 °C for 10 min, the supernatant was discarded, and the cell pellet was resuspended and cultured in pre-warmed Dulbecco’s modified Eagle’s medium (Gibco, Grand Island, NY, United States) supplemented with 10% fetal bovine serum (Gibco, Gaithersburg, MD, United States) and 1% penicillin–streptomycin (Gibco, Grand Island, NY, United States). Cultures were maintained at 37 °C in a humidified atmosphere with 5% CO_2_. Third-passage ADSCs were used for characterization and tri-lineage differentiation. Adipogenic, osteogenic, and chondrogenic differentiation were induced using specific conditional differentiation media, as previously described (Yao et al., 2021). To examine adipogenic differentiation, ADSCs were cultured in the adipogenic induction medium for 20 days, followed by Oil Red O-staining to visualize lipid droplets. To assess osteogenic differentiation, ADSCs were cultured in the osteogenic or chondrogenic induction medium for 21 days. Calcium deposition in bone nodules was assessed using alizarin red dye and an alkaline phosphatase staining kit. For chondrogenic differentiation analysis, ADSCs were cultured in the chondrogenic induction medium for 21 days. Chondrogenic lineage differentiation was confirmed by toluidine blue-staining. All positively stained areas were examined under a microscope with 200× magnification (Carl Zeiss, Oberkochen, Germany). The expression levels of various ADSCs’ surface markers, including CD34, CD45, CD90, CD105, and CD10 (Abcam, Cambridge, United Kingdom), were assessed via flow cytometry, and the purity of the ADSCs was consistently >90%.

### *In vivo* tracing of ADSCs

For *in vivo* tracing experiments, rat ADSCs were transduced with specially designed lentiviral vectors containing both green fluorescence protein (GFP) and luciferase reporter genes. ADSCs transfected with this vector could be detected *in vitro* using fluorescence microscopy via GFP expression and *in vivo* using the *In Vivo* Imaging System (IVIS) via luciferase expression (GFP/luciferase-ADSCs). Next, 10^7^ GFP/luciferase-ADSCs were transplanted into female SD rats and tracked *in vivo* using IVIS. The detailed transplantation procedure was as follows: the surface of the medial rectus femoris on the left thigh (irradiation site) was marked using a marker. Then, GFP/luciferase-ADSCs were resuspended in 100 μL of PBS and divided into 25 μL per injection. A volume of 25 μL was injected at four points surrounding the marked site: upper, lower, left, and right.

### Histological examinations of hematoxylin–eosin and Masson’s trichrome staining

Skeletal muscle tissues from each group were fixed in 4% paraformaldehyde for 24 h, embedded in paraffin, and sectioned at 4 μm thickness. The sectioned lamellae were stained with H&E (ServiceBio, GP1031) and Masson’s trichrome (ServiceBio, GP1032) following the manufacturer’s protocols. The tissues were analyzed, and images were acquired using an Axio Scope A1 Inverted Microscope (Carl Zeiss, Oberkochen, Germany). The percentage of skeletal muscle fibrosis was quantified using Image-Pro Plus 6.0 software. All visual fields in each tissue section were analyzed. First, the areas of blue-stained collagen fibers and the total tissue area were measured. Then, the percentage of fibrosis was calculated as the ratio of the collagen fiber area to the total area.

### Electron microscopy

Samples prepared for transmission electron microscopy analysis were fixed in 2.5% glutaraldehyde and washed with PBS several times to remove impurities. Post-fixation was carried out for 2 h using 1% osmium tetroxide. Following fixation, the samples were dehydrated through a graded ethanol series followed by propylene oxide treatment, and then they were gradually embedded in Epon 812 resin. Ultrathin sections (50-nm thickness) were obtained and double-stained with 3% uranyl acetate and lead citrate for 15 min. The ultrastructure of skeletal muscle cells, including organelles and vascular structures, was observed and recorded using a transmission electron microscope (FEI, Hillsboro, OR, United States).

### Real-time polymerase chain reaction

Total RNA was extracted from muscle tissues using the SteadyPure RNA Extraction Kit (Accurate Biology, Changsha, China). For each sample, 1 µg of total RNA was reverse-transcribed into cDNA using the Evo M-MLV Reverse Transcription Kit (Accurate Biology, Changsha, China). RT-PCR was performed on the LightCycler 96 system (Roche, Basel, Switzerland) using the Hieff® qPCR SYBR Green Master Mix (No Rox) (Yeasen, Shanghai, China). Gene expression levels were calculated using the comparative cycle threshold (Ct) method, with normalization to B2M. The housekeeping gene *Gapdh* was selected as an internal reference, and relative Ct values were calculated using the average data of triplicate experiments. The primer sequences used in this study are listed in Table 1.

**TABLE 1:** Primer sequence of the marker genes.

Gene name	Forward primer (5′–3′)	Reverse primer (5′–3′)
GAPDH	AGGTCGGTGTGAACGGATTTG	TGTAGACCATGTAGTTGAGGTCA
PAX7	GAGTATAAGAGGGAGAACCCCG	TTGATTCTGAGCACTCGGCTAA
MyoD	GCTCTGATGGCATGATGGATTAC	CTATGCTGGACAGGCAGTCG
MyoG	ACTACCTTCCTGTCCACCTTCA	AGGCCTCATTCACTTTCTTGAG
Mrf4	ACAGCTACAAACCCAAGCAAGA	CTTGCTCCTCCTTCCTTAGCAG
Myf5	TCTGATGGCATGCCTGAATGTAA	AAGGAGCTCTTATCTGAAGCACA

### Terminal deoxynucleotidyl transferase dUTP nick end labeling staining

Apoptotic cells in irradiated muscle tissues were detected using the terminal deoxynucleotidyl transferase dUTP nick end labeling (TUNEL) apoptosis assay kit (G1501; ServiceBio, Wuhan, China), as described in our previous study (Sheng et al., 2019). TUNEL-positive cells were counted under an inverted fluorescence microscope (Carl Zeiss, Oberkochen, Germany), and the percentage of TUNEL-positive cells was calculated.

### Statistical analysis

All data are presented as the mean ± standard error of the mean. Statistical analyses were performed using SPSS (version 28.0; SPSS, Chicago, Illinois, United States). Differences between the two groups were analyzed using the Student’s t-test. For comparisons among multiple groups, one-way analysis of variance was applied. A *p*-value <0.05 was considered statistically significant.

## Results

### Extraction and characterization of primary ADSCs

**FIGURE 1:**
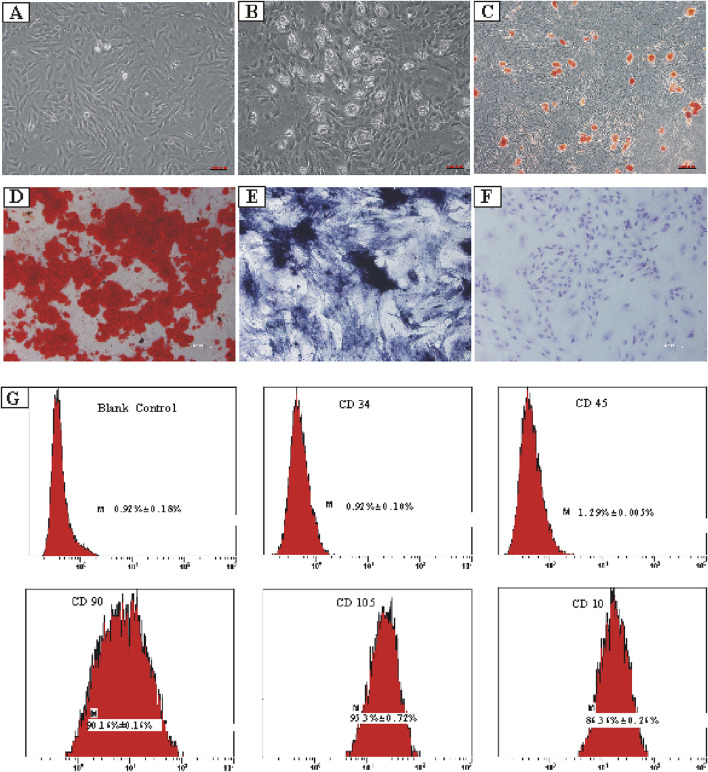
Isolation and lineage tracing of ADSCs, including tri-lineage differentiation and flow cytometry analysis. **(A)** ADSCs separated from superficial subcutaneous adipose tissue of male rats. **(B)** Differentiation of the ADSCs into adipocytes. **(C)** Precipitation of oil droplets in the differentiated ADSCs detected using oil red O-staining. **(D)** Osteogenic differentiation of ADSCs confirmed by calcium deposition using alizarin red-staining. **(E)** Osteogenic differentiation of ADSCs verified using alkaline phosphatase-staining. **(F)** Chondrogenic differentiation of ADSCs visualized using toluidine blue-staining. **(G)** Expression percentages of various ADSCs’ surface markers. CD34: 0.92% ± 0.10%, CD45: 1.29% ± 0.005%, CD90: 90.16% ± 0.16%, CD105: 97.53% ± 0.72%, and CD10: 86.36% ± 0.26%.

ADSCs were extracted and isolated from the superficial subcutaneous adipose tissue of male rats. The cultured primary ADSCs exhibited a relatively homogeneous, spindle-shaped morphology and demonstrated robust growth (Figure 1A). Notably, ADSCs displayed strong adipogenic, osteogenic, and chondrogenic differentiation potential when cultured in the specific induction differentiation medium (Figures 1B–F). Subsequently, a flow cytometer was used to confirm the surface markers of ADSCs *in vitro*, and the results showed high expression levels of CD90 (90.16% ± 0.16%), CD105 (97.53% ± 0.72%), and CD10 (86.36% ± 0.26%), along with low expression levels of CD34 (0.92% ± 0.10%) and CD45 (1.29% ± 0.005%) (Figure 1G).

### Cell tracing of ADSCs

**FIGURE 2:**
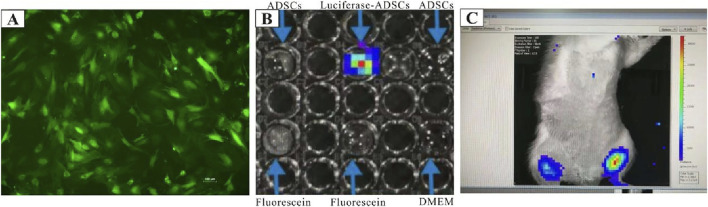
Cell tracing of ADSCs *in vitro* and *in vivo*. **(A)** GFP/luciferase-ADSCs observed under fluorescence microscopy *in vitro.* **(B)** *In vitro* bioluminescence imaging of GFP/luciferase-ADSCs using the IVIS system. **(C)** *In vivo* tracking of GFP/luciferase-ADSCs following injection into rats. High-intensity fluorescence signals were detected with IVIS at 2 h post-injection, compared with the contralateral trials.

To assess the post-transplant survival of ADSCs *in vivo*, GFP/luciferase-ADSCs were harvested. First, the expression of GFP in the transduced ADSCs was confirmed *in vitro* using fluorescence microscopy, where most cells exhibited green fluorescence (Figure 2A). We also observed cells expressing firefly fluorescence *in vitro* using IVIS, and intense fluorescence was detected (Figure 2B). For *in vivo* tracking, GFP/luciferase-ADSCs were intramuscularly injected into female rats, and the luciferase signal was monitored via IVIS at 2 h post-injection; an intense, localized fluorescence signal was detected within the injection site (Figure 2C).

### ADSC treatment attenuates muscle fibrosis induced by radiation

**FIGURE 3:**
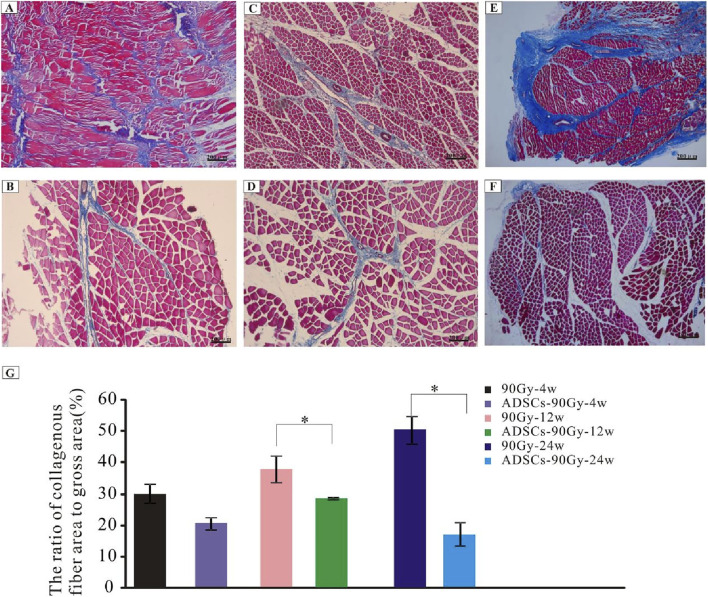
Degree of fibrosis in the control and ADSC-treated groups. Masson’s trichrome-staining of irradiated muscle tissues from rats in the control group: **(A)** 90 Gy-4 w, **(C)** 90 Gy-12 w, and **(E)** 90 Gy-24 w and from rats in the ADSC-treated group: **(B)** ADSCs-90 Gy-4 w, **(D)** ADSCs-90 Gy-12 w, and **(F)** ADSCs-90 Gy-24 w. **(G)** Quantification of collagen content shows a reduction in the percentage of skeletal muscle collagenous fibers in the ADSC-treated groups than that in the control group. *p < 0.05.

To ascertain the extent of muscle fibrosis in RIF rats, Masson’s trichrome staining method was carried out in the control and ADSC-treated groups. Increased collagen deposition in muscle tissues was observed over time (Figures 3A, C, E); however, this collagen accumulation could be reversed by ADSC treatment (Figures 3B, D, F). Although the collagen fibers did not significantly decrease by ADSC treatment at the acute injury stage, the percentage of skeletal muscle collagenous fibers showed a pronounced reduction after treatment at the advanced stage (Figure 3G).

### ADSCs alleviate radiation-induced injuries in muscles

**FIGURE 4:**
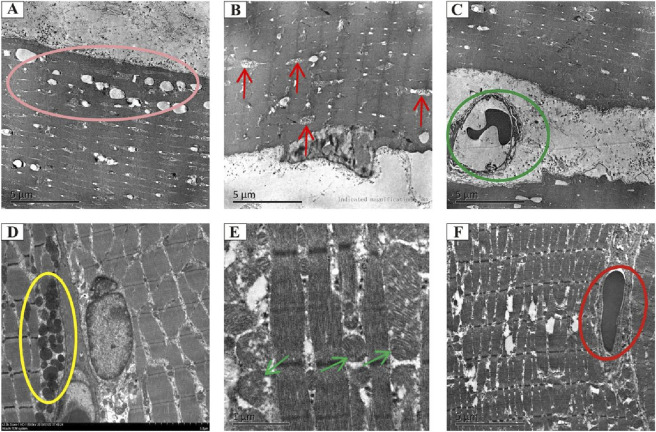
Transmission electron microscopy analysis of the morphology and microstructure of rat muscle tissues. Representative electron micrographs showing the ultrastructure of muscle tissues in the model cohort (90 Gy-4 w) **(A–C)** and the ADSC-treated cohort (ADSCs-90 Gy-4 w) **(D–F)**. The pink circle indicates vacuolization and edema in mitochondria **(A)**. Red arrows indicate mitochondrial cristae loss and vacuolization **(B)**. Green circles indicate irregular vasculature **(C)**. Yellow circles show mitochondria with no apparent structural abnormalities **(D)**. Green arrows indicate mitochondria with no obvious vacuolization **(E)**. Blue circle marks vascular endothelium with no obvious swelling **(F)**.

To investigate the intricate architecture damage *in vivo*, the morphological and ultrastructural features of muscle tissues obtained from each group were characterized using transmission electron microscopy. Vacuolization and edema in the mitochondria, dissolved myofilaments, and vascular endothelial swelling were observed in the RIF model cohort at 4 weeks post-radiation (Figures 4A–C). However, these disorders were partially or completely rescued by ADSC transplantation (Figures 4D–F).

### ADSCs promote activation, proliferation, and differentiation of SCs *in vivo*

**FIGURE 5:**
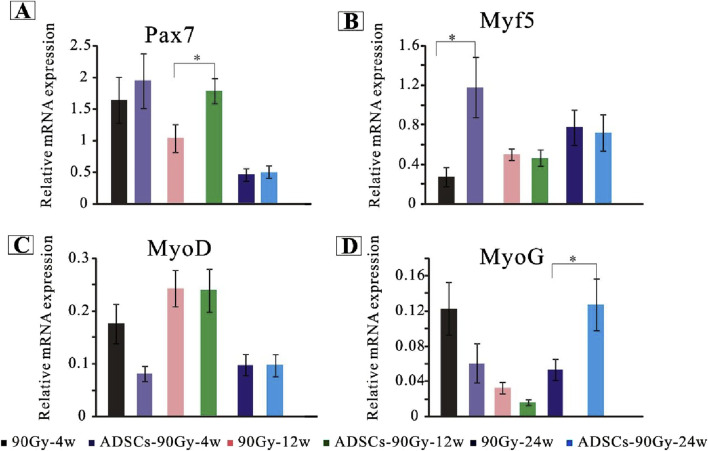
ADSCs enhance SC activation in response to radiation-induced muscle damage. Quantitative polymerase chain reaction analysis of gene expression levels for key markers of SC activation, proliferation, and differentiation in the control and ADSC-treated groups, including *Pax7* **(A)**, *Myf5* **(B)**, *MyoD* **(C)**, and *MyoG* **(D)**.*p < 0.05.

To examine the mechanism of ADSC-induced SC stabilization, activation, and myogenic processing, the relative gene expression levels were quantified using RT-PCR. Myogenic-associated transcription factors, including Pax7, Myf5, MyoD, and MyoG, were analyzed as markers of SC activation and myogenesis. Our results revealed that Pax7 was significantly upregulated at 12 weeks post-irradiation in the ADSC group compared with that in the control group (Figure 5A). Although the gene expression of MyoD did not differ in the irradiated muscles of each group across the whole trial epochs (Figure 5C), Myf5 expression elevated drastically at 4 weeks post-irradiation in the ADSC group (Figure 5B). Moreover, the expression of the myogenic marker MyoG was increased in the ADSC-treated group at 24 weeks post-irradiation (Figure 5D). Together, our results provide new insights regarding ADSC therapy targeting radiation-induced muscle injury.

### ADSCs facilitate central nuclear translocation in RIF rat muscle fibers

**FIGURE 6:**
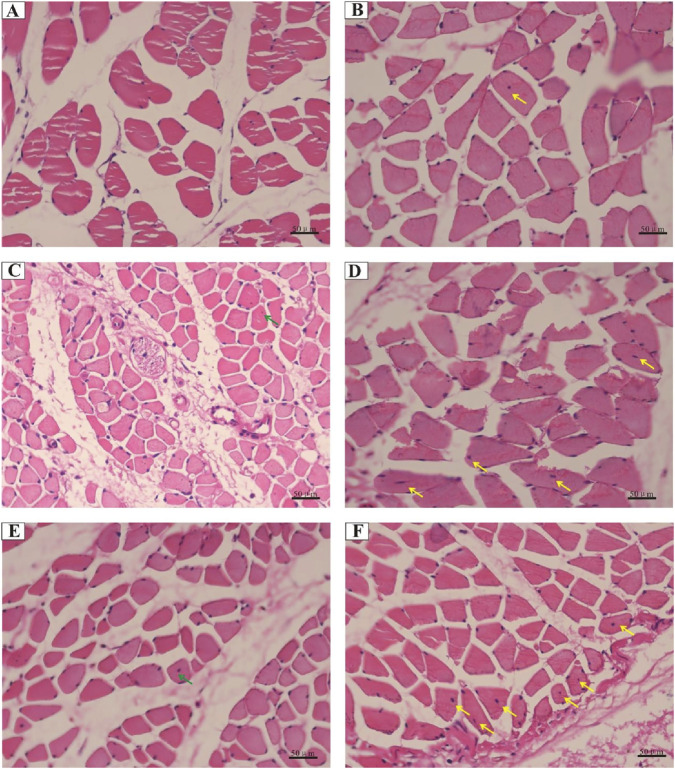
Central nuclear translocation in muscle fibers following radiation exposure. Representative histology images of hematoxylin and eosin-stained muscle sections from rats of the control groups: **(A)** 90 Gy-4 w, **(C)** 90 Gy-12 w, and **(E)** 90 Gy-24 w and from rats of ADSC-treated experimental groups: **(B)** ADSCs-90 Gy-4 w, **(D)** ADSCs-90 Gy-12 w, and **(F)** ADSCs-90Gy-24 w. Green arrows indicate a limited number of centrally nucleated myofibers in the control groups, while yellow arrows indicate highly centrally nucleated myofibers in the ADSC-treated groups.

Considering the important role of central nuclear migration in muscle regeneration, we performed H&E-staining to confirm nuclear positioning *in vivo* and quantify it. We transplanted ADSCs into the femoris muscle of normal rats and found that the muscle structure remained normal, and no central nuclei appeared (data not shown). Comparison to the control littermates, irradiated rats appeared susceptible to central nuclear translocation in muscle fibers, ranging from limited to intermediate levels in the ADSC groups (Figure 6). At 4 weeks post-irradiation, skeletal muscle experienced a progressive deterioration in fiber alignment, with no apparent centralized nuclei; instead, increased proportions of myofibers with central nucleation were determined within the ADSC group (Figures 6A, B). Notably, limited centrally nucleated myofibers appeared at 12 and 24 weeks post-irradiation in the mammalian models (Figures 6C, E), and the centrally located nuclei exhibited high susceptibility to ADSC treatment with the extent of time (Figures 6B, D, F). Combined, these results provide strong support for our hypothesis that ADSCs may promote muscle regeneration in the RIF model rats.

### ADSCs inhibit apoptosis in irradiated muscle tissue

**FIGURE 7:**
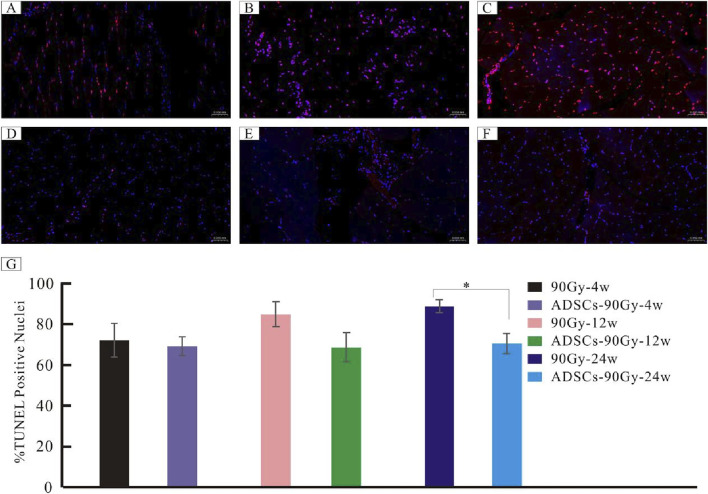
Detection of apoptotic cells in irradiated muscle tissues. Representative TUNEL-stained images are shown for the control groups: **(A)** 90 Gy-4 w, **(B)** 90 Gy-12 w, and **(C)** 90 Gy-24 w and the ADSC-treated experimental groups: **(D)** ADSCs-90Gy-4w, **(E)** ADSCs-90Gy-12 w, and **(F)** ADSCs-90Gy-24 w. TUNEL-positive nuclei are stained red. The percentage of apoptotic cells was significantly higher in the 90 Gy-24 w control group than that in the ADSCs-90Gy-24 w group. Data are presented as the mean ± SEM. *p < 0.05 compared with the respective control group. **(G)** The Percentage of apoptotic cells was significantly higher in the 90Gy-24 w control group than that in the ADSCs-90Gy-24 w group.

Representative images of TUNEL‐positive apoptotic cells from each group are shown in Figures 7A–F. A large number of apoptotic cells were found in the control 90 Gy-4 w (A), 90 Gy-12 w (B), and 90 Gy-24 w (C) groups, but only a few apoptotic cells were found in the ADSCs-90 Gy-4 w (D), ADSCs-90 Gy-12 w(E), and ADSCs-90 Gy-24 w(F) groups. In contrast to the control group, although the number of apoptotic cells slightly decreased in the ADSC-treated group at 4 and 12 weeks, the differences were not statistically significant, but the difference at 24 weeks was statistically significant (Figure 7G). These findings revealed that ADSCs inhibit apoptosis in irradiated muscle *in vivo*.

## Discussion

RIF is a severe advanced complication of radiotherapy, especially in patients with head-and-neck tumors or breast cancer subtypes (Lennox et al., 2002; van Geel et al., 2011). Although various treatment strategies are available to manage RIF, the therapeutic efficacy of existing modalities remains limited (Okunieff et al., 2004; Wang et al., 2022; Krejbich and Birringer, 2022), providing renewed impetus for the exploring novel therapeutic approaches. In this study, we demonstrated that ADSCs ameliorated muscle tissue injuries by reducing collagen fibrillogenesis; inhibiting apoptosis; and promoting SC activation, proliferation, and differentiation *in vivo*. These findings provide fundamental evidence for the clinical therapeutic potential of ADSCs in RIF.

ADSCs are highly promising for multipotent stem cell-based therapies due to their easy accessibility, cost-effectiveness, and high proliferation. Although no unique single-cell surface marker characterizes ADSCs, they exhibit functional characteristics similar to mesenchymal stem cells, such as adipogenic, osteogenic, and chondrogenic differentiation capability *in vitro*. In addition to tri-lineage differentiation, ADSCs express surface markers such as CD90, CD105, and CD10, which is consistent with our previous findings (Yao et al., 2021). In this study, we characterized ADSCs using previously described ways of representation.

ADSCs represent a new therapeutic strategy in musculoskeletal diseases. Over the past several decades, numerous growth factors secreted by ADSCs, including IGF-1, TGF-β1, bFGF, VEGF, and hepatocyte growth factor, have been shown to be associated with growth *in vivo*, providing robust evidence for their role in muscle repair (Rivera-Izquierdo et al., 2019). Radiation exposure induces long-term muscle atrophy and fibrosis (Collao et al., 2023). In our previous study, we established an RIF rat model (Zhou et al., 2018) and observed SC activation; however, this activation-related muscle regeneration was not sufficient to counteract fibrosis formation (Zeng et al., 2022). Ni et al. (2014) reported that ADSC transplantation repaired radiation-induced skeletal muscle injury in New Zealand white rabbit models, which was associated with the upregulation of VEGF and bFGF. Wang et al. (2014) demonstrated that transplantation of bone marrow stromal cells overexpressing VEGF enhanced muscle repair in radiation-injured rat models. In addition, Rybalko et al. (2017) reported that co-culturing ADSCs with macrophages ameliorated the functional decline and reperfusion injury in typical peripheral artery disease, resulting in enhanced skeletal muscle regeneration. Gastrocnemius muscular atrophy caused by irretrievable resection and retraction of the sciatic nerve in a programmed process could be rescued through diffuse intramuscular injection of human ADSCs in mice (Qu et al., 2022; Schilling et al., 2019). Numerous clinical trials utilized ADSCs to treat various diseases (Lee et al., 2019; Li et al., 2023; Fujita et al., 2023; Iglesias et al., 2023), and we anticipate future clinical applications targeting muscle fibrosis and atrophy. In our study, ADSCs alleviated collagen fiber formation, inhibited apoptosis, promoted SC proliferation, and enhanced myoblast differentiation and muscle regeneration in irradiated muscle tissue *in vivo*. However, the mechanisms underlying these therapeutic effects remain to be elucidated. In the future, our findings may serve as a foundation for exploring the underlying cellular mechanisms of ADSC-based treatment.

Under physiological conditions, muscle regeneration and degradation are maintained in a dynamic equilibrium. However, under pathological conditions, this homeostasis is disrupted, leading to excessive deposition of fibrillar collagen in damaged muscle tissue (Zeng et al., 2022). The balance between collagen deposition and muscle fiber regeneration is complex and poorly regulated. Recent advances in biogenetics and cellular biology have highlighted that mitochondrial metabolism plays a crucial role in apoptosis, a form of programmed cell death (Schapira, 2012). Apoptosis of oligodendrocytes was significantly increased in a rat model of radiation-induced diffuse brain injury (Sano et al., 2000). In our previous work, we identified apoptosis as a key factor in a radiation-induced dermatitis model (Yao et al., 2021). Our current results showed a high level of apoptotic cells and mitochondrial abnormalities in irradiated muscle tissue, which is inconsistent with prior findings. However, this could be alleviated following ADSC treatment. Traditionally, central nuclear migration in nascent muscle fibers and SC activation are both regarded as sources of muscle regeneration (Srikuea and Hirunsai, 2016). In our study, although ADSCs demonstrated promising potential for treating RIF, their therapeutic effects require further improvement. Future work will focus on improving the regenerative capacity of ADSCs to more effectively promote muscle repair and treat RIF.

## Conclusion

Collectively, our findings provide evidence of the therapeutic potential of ADSCs in treating chronic RIF *in vivo*. Collagen deposition showed an apparent decrease in the ADSC-treated group compared with the control group. Additionally, ADSCs promoted SC activation, proliferation, differentiation, and central nuclear formation in muscle cells. These results suggest that ADSCs are a promising candidate for repairing radiation-impaired muscle in tissue engineering.

## Data Availability

The raw data supporting the conclusions of this article will be made available by the authors, without undue reservation.
